# Pure neural leprosy in a child: case report and diagnostic challenges

**DOI:** 10.1590/0037-8682-0158-2025

**Published:** 2026-03-02

**Authors:** Hortência Aparecida dos Reis Santana, Isabela Pedra Diamantino, Jean Carlos de Araújo Arruda, Pedro Alysson Mota da Silva, Gabrieli Souza dos Santos, Jonatas César Matos de Oliveira, Vanessa Oliveira de Souza, Clécio Ribeiro Costa, Andrea Pereira Macke, Karen Nepomuceno Sá Teles, Fernando do Prado Vieira, Filipe Rocha Lima, Jonilson Berlink Lima

**Affiliations:** 1 Universidade Federal do Oeste da Bahia, Centro das Ciências Biológicas e da Saúde, Barreiras, BA, Brasil.; 2 Centro de Saúde Leonídia Ayres de Almeida, Programa de Controle da Hanseníese, Barreiras, BA, Brasil.; 3 Universidade de São Paulo, Faculdade de Medicina de Ribeirão Preto, Hospital das Clínicas, Divisão de Dermatologia, Departamento de Clínica Médica, Ribeirão Preto, SP, Brasil.; 4 Universidade de São Paulo, Faculdade de Medicina de Ribeirão Preto, Departamento de Bioquímica e Imunologia, Ribeirão Preto, SP, Brasil.; 5 Núcleo de Agentes infeciosos e Vetores (NAIVE), Barreiras, BA, Brasil.

**Keywords:** Pure neural leprosy, Leprosy reaction, Diagnosis

## Abstract

Pure neural leprosy (PNL) is infrequent and manifests exclusively in the peripheral nerves without skin involvement, making the diagnosis more complex. We report the case of a 10-year-old child with muscle atrophy and sensory loss, diagnosed through clinical evaluation, grade 2 disability, and a positive anti-PGL-I test. The slit-skin smear (SSS) was negative for acid-fast bacilli. After 12 months of multidrug therapy, esthesiometric sensitivity improved; however, neurological deficits persisted, requiring anti-inflammatory treatment and physiotherapy. This case highlights the importance of early diagnosis and treatment for preventing disabilities. Tools, such as anti-PGL-I tests and imaging, are crucial, particularly in resource-limited settings.

## INTRODUCTION

Pure neural leprosy (PNL) was first clinically classified by the Indian Association of Leprology in 1955. Epidemiological data indicate that PNL represents only 3-10% of leprosy cases, commonly manifesting as neurological deficits without skin lesions and negative bacilloscopy in slit-skin smears (SSS)^1^. 

It is more common in men and in the 15- to 30-year age group, with cases in children being rare^1^. Symptoms such as sensory and motor impairments, nerve thickening, and altered sensitivity characterize PNL. The diagnosis of PNL tends to be delayed because, unlike patients with skin lesions, these individuals generally do not receive early dermatological referral^2^.

However, its prevalence may be underestimated because of its slow and nonspecific progression. Certain studies have suggested that leprosy remains underdiagnosed in Brazil, even in areas with low endemicity. These findings corroborate that, even in supposedly non-endemic areas or areas with low detection rates, a significant hidden burden of the disease may exist, reinforcing the relevance of the present study^3^.

PNL can be characterized by a set of symptoms, such as thickening of peripheral nerves; reduced sensitivity; anesthetized areas; tingling; numbness and pain; swelling of the face, hands, and feet; burning sensation; motor deficits; autonomic nerve dysfunction; and dysautonomia^2^ ^,^ ^3^. Diagnosis is complex and requires a complete clinical evaluation, including nerve palpation and sensory and motor function tests, aided by additional examinations. Currently, nerve biopsies with histopathological and molecular analyses (polymerase chain reaction [PCR]), as well as high-resolution ultrasound, are considered the most accurate tools for confirmation^1^. Furthermore, tests, such as electromyography and serology for the PGL-I antigen, can guide clinical suspicion or rule out other neuropathies. However, none of these tests alone can confirm the diagnosis of PNL^4^ ^,^ ^5^.

This condition presents as a rare clinical presentation with high diagnostic complexity, which hinders early detection^4^. Children with PNL may present with grade 2 disabilities at the time of diagnosis, highlighting the insidious and progressive course of the neuropathy^6^. In addition, this diagnostic challenge is observed in non-endemic areas, as reported by Payne et al. (2015), in which a pediatric patient was diagnosed with PNL only after a neurophysiological assessment and nerve biopsy in the context of severe ulnar neuropathy^7^.

However, the number of published case studies reporting PNL in children remains limited, demonstrating that the proper diagnosis and management of these patients remains challenging in clinical practice. Adequate knowledge of PNL in this population is essential for the early diagnosis and prevention of disabilities.

## CASE REPORT

A 10-year-old boy was referred to the Leprosy Program at the Leonídia Ayres Health Center in Barreiras, BA, in June 2023 for evaluation of primary neural leprosy (PNL). The patient was referred for neurological complaints, including numbness in the limbs. Bacilloscopy examination was negative for acid-fast bacilli (SSS), reinforcing the need for thorough clinical and neurological investigations.

**FIGURE 1:**
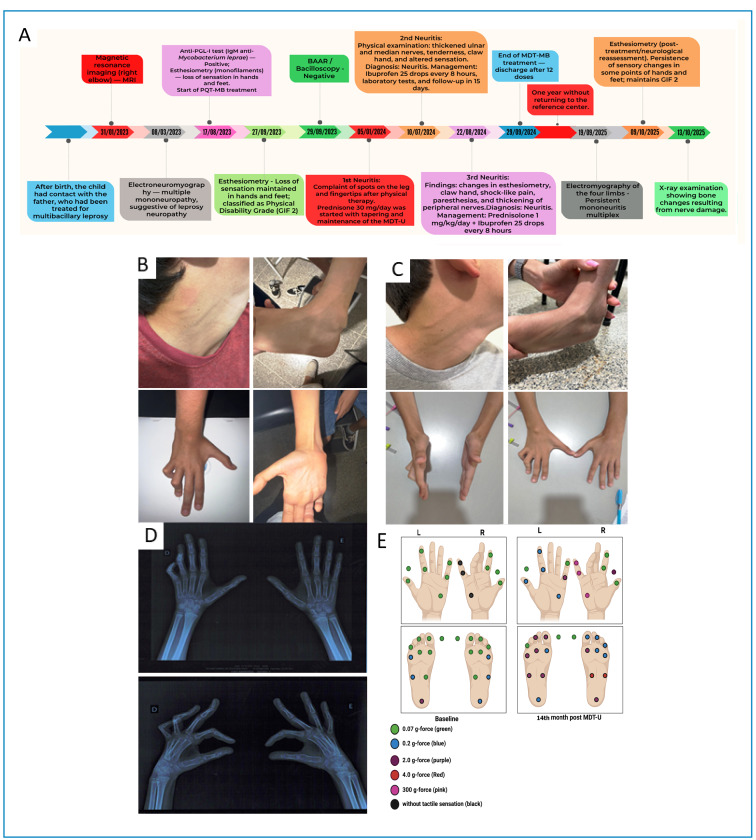
Clinical, neurological, and functional evolution of the patient with leprosy. **(A)** Timeline illustrating the patient’s clinical follow-up from the initial diagnosis to the most recent evaluation. **(B)** Thickening of the left auricular and left sural nerves, associated with right ulnar claw deformity observed in 2024. **(C)** Clinical reassessment in October 2025, demonstrating partial persistence of neurological and motor abnormalities. **(D)** Hand radiographs obtained in October 2025. **(E)** Comparison of esthesiometry results before the initiation of multibacillary multidrug therapy (MB-MDT) and after 14 months of treatment, demonstrating improved tactile sensitivity in the right hand and worsening sensitivity in the feet.

During anamnesis, it was reported that a few months after birth, the patient had contact with a close relative (father), a former patient with leprosy. However, it was not possible to determine the period of cohabitation before treatment began, nor was contact tracing screening conducted. In the initial medical care, physical examination revealed thenar and hypothenar atrophy of the right hand, deformity of the 4th and 5th digits in the claw, and loss of thermal and pain sensitivity in the palmar and plantar regions (Figure 1E).

The patient did not present with skin lesions. The clinical findings focused mainly on the asymmetry observed in the esthetics of the hands and feet and on the electroneuromyography (ENMG) findings characteristic of leprosy, consistent with focal and asymmetric multiple mononeuropathy with sensory loss in the neural pathway as the cardinal sign of the disease. This diagnostic pattern is in line with a study by Frade *et al.* (2022), which highlighted the relevance of altered tactile sensitivity patterns assessed by Semmes-Weinstein monofilaments in the diagnosis and monitoring of leprosy^8^.

At the initial consultation, the patient presented previous examinations, including an ENMG and a magnetic resonance imaging (MRI) scan of the right elbow, which revealed mild bone edema in the humerus. EMG allows for the definition of patterns of peripheral neural involvement and early detection of peripheral leprosy disease neuropathy^8^, which, in this case, revealed predominantly axonal, asymmetrical, and sensorimotor neuropathy involving multiple nerves in the upper and lower limbs. Examination revealed moderate sensorimotor axonal neuropathy of the left median nerve, marked sensorimotor axonal neuropathy of the right radial nerve, marked sensorimotor axonal neuropathy of the left radial nerve, marked bilateral axonal neuropathy of the superficial fibular nerves, marked axonal neuropathy of the left tibial nerve, and marked sensorimotor axonal neuropathy of the sural nerves bilaterally. 

These ENMG findings indicated a pattern of multiple mononeuropathies (multiple mononeuritis). This corroborates data from studies that demonstrate that the nerves most frequently affected in leprosy, according to ENMG evaluation, are the ulnar, superficial radial, sural, superficial fibular, and sensory tibial nerves, and that the common fibular and median nerves are the least frequently affected. The tibial nerve is considered the most significantly affected in leprosy^8^.

The physical examination of the ulnar nerve was performed according to the protocol described in the Simplified National Neurological Assessment Manual^4^. In summary, the patient’s hand was supported by the examiner with the arm relaxed and the elbow flexed. Using the free hand, the examiner palpated the cubital tunnel to locate the ulnar nerve and assessed its characteristics, including spontaneous pain along its path, sensation of pain or shock on palpation, symmetry, size, shape, consistency, and the presence of nodules. Subjective complaints of pain and shock related to the patient were also recorded.

Based on the clinical and laboratory findings, differential diagnoses such as traumatic injuries, hereditary neuropathies including Charcot-Marie-Tooth disease, and metabolic causes such as diabetes mellitus and vitamin deficiencies were considered and ruled out. This diagnostic exclusion was corroborated by a detailed medical history and evaluation of laboratory tests, which showed hematological parameters within the reference values defined by the clinical analysis laboratory.

Hemoglobin at 13.8 g/dL, hematocrit at 39.8%, and leukocyte count of 5,300/mm³, with segmented neutrophils within normal limits, indicated appropriate bone marrow function and the absence of acute infectious processes. Furthermore, the platelet count (272,000/mm³) was within the expected normal range. Inflammatory markers remained stable, with a C-reactive protein level of < 6 and an erythrocyte sedimentation rate of 18 mm, suggesting no active systemic inflammation.

Regarding micronutrient metabolism, the levels of vitamin D (31.6 ng/mL), ferritin (39.70 ng/mL), and serum iron (60.1 µg/dL) are within values consistent with adequate reserves and preserved iron and vitamin homeostasis. Activities of liver enzymes such as AST (23 U/L), ALT (31 U/L), and GGT (15 U/L) were within normal limits, indicating preserved hepatocellular function. A negative ANA test ruled out significant autoimmune activity, and a fasting glucose level of 78 mg/dL was consistent with normal carbohydrate metabolism.

Overall, the combined clinical and laboratory data did not indicate significant systemic abnormalities, supporting the exclusion of relevant metabolic, autoimmune, infectious, or hematological etiologies.

The initial sensitivity assessment using esthesiometry revealed a complete absence of response to monofilaments in the region innervated by the right ulnar nerve, indicating a physical disability grade of 2 according to the World Health Organization criteria. The other areas showed normal responses. In addition to sensory loss, an ulnar claw deformity was observed in the right upper limb, consistent with a similar degree of disability. These results, along with the neural thickening detected in physical examination and complaints of numbness, reinforced the clinical suspicion of primary neural leprosy with ulnar nerve involvement.

During the clinical evaluation, the patient did not present with skin lesions but exhibited a BCG vaccine scar, claw fingers, and bone resorption in his right hand (Figure 1B). The anti-phenolic glycolipid-I (PGL-I) screening test was positive, confirming contact with the *M. leprae*. Therefore, multibacillary (MB; 12 months) multidrug therapy (MDT) was initiated, and general guidelines were provided to the family according to the Clinical Protocol and Therapeutic Guidelines for Leprosy of the Brazilian Ministry of Health.

After 12 months of treatment, the patient showed improvement in pain symptoms; however, nerve thickening and sensitivity alterations persisted. In addition, new signs of lower-limb involvement were identified (Figure 1C). 

Since the beginning of treatment, the patient presented with recurrent episodes of neuritis characterized by pain and neural thickening, most evident in the right ulnar and radial nerves. The first episode of neuritis occurred in January 2024 and was treated with prednisone (30 mg) and a tapering regimen. In July 2024, a second episode was recorded, and ibuprofen and physiotherapy were prescribed. A third episode was documented in August 2024 and was treated with prednisolone (1 mg/kg/day) and ibuprofen combined with physiotherapy sessions.

After completing multidrug therapy (MDT-MB) in September 2024, the patient remained absent from the referral service for approximately 1 year, returning only in October 2025 for reassessment. Radiographs of the affected limbs and new esthesiometry were requested and performed on October 9, 2025. The most recent simplified neurological analysis showed improved sensitivity in the areas innervated by the right ulnar and median nerves, with a response to pink monofilaments (300 gf), but worsened sensitivity in the lower limbs, especially in the regions innervated by the fibular and tibial nerves, which began to respond only to weaker filaments. Furthermore, the left upper limb had worsened, with thickening and shock pain in the median, ulnar, and radial nerves (Figure 1A). In addition, radiographs obtained during the patient's return visit on October 13, 2025, in anteroposterior and oblique projections revealed deformities of the fourth and fifth fingers of the right hand, characterized by flexion at the proximal interphalangeal joints, without evidence of bone resorption on the corresponding articular surfaces (Figure 1D). The remaining bone structures presented a morphology and density compatible with the patient's age range, as well as preserved joint spaces and soft tissues without relevant densitometric alterations.

Clinical and functional findings indicated a partial improvement in neural function in the upper limbs, in contrast to the progression of sensory changes in the lower limbs, consistent with the chronic evolution of multineural leprosy neuropathy with residual sequelae.

Important Brazilian studies on neurological assessment and the proposition of diagnostic strategies for PNL provide evidence that reinforces our finding: “The presence of neural thickening and demyelinating impairment favors the diagnosis of leprosy, and patients in this study’s PNL group presented an asymmetric multiple mononeuropathy.” Therefore, careful clinical evaluation is mandatory to corroborate the diagnosis of leprosy in the presence of the described morphological or neurophysiological changes. Furthermore, documenting the presence of bacilli, even in peripheral nerve biopsy samples, represents a major challenge for early diagnosis, especially in cases of primary neural leprosy. Therefore, new tools are required, especially for corroborating epidemiological and clinical context^9^.

In the present case, the patient had a clinical picture compatible with peripheral neuropathy, showing neural thickening on physical examination, positive anti-PGL-I serology, and strong epidemiological evidence, given the endemic region in which he resides and the direct and prolonged intradomiciliary contact with the parent previously diagnosed with multibacillary leprosy. These elements, when analyzed together, supported the diagnostic hypothesis and justified therapeutic decisions. In addition, sensory evaluation was performed using esthesiometric tests with Semmes-Weinstein monofilaments applied in a standardized manner before and after treatment with multidrug therapy^8^. An improvement in response to tactile stimuli was observed in post-treatment reassessment, which was interpreted as an additional clinical element reinforcing the diagnosis, although it was admittedly nonspecific and not used as an isolated diagnostic criterion. These findings can help discriminate leprosy from other neuropathy etiologies by revealing asymmetry, irregular thickening, and demyelination. In addition, epidemiological and immunological results were positive, which indicates that the bacillus has successfully penetrated the circulatory system and represents a relative risk almost six times higher for disease occurrence, corroborating the leprosy neuropathy diagnosis^1^. 

Considering that the patient was pediatric, a nerve biopsy was not performed because of the invasive nature of the procedure and the clinical context at the time of the therapeutic decision. Furthermore, the patient had already started treatment with polychemotherapy, a condition that should be carefully considered when indicating a possible late biopsy, as specific therapy can reduce the diagnostic yield both histopathologically and molecularly, particularly in cases known to be paucibacillary.

## DISCUSSION

Diagnosis of PNL is often difficult and delayed, resulting in disability at the time of presentation^7^. Nerve biopsy can identify typical leprosy changes, such as perineural inflammation, fiber disorganization, and granulomatous infiltration, and is considered the gold standard for detecting PNL in several cases. High-resolution ultrasound is specifically used in PNL, as it has proven valuable in visualizing increased blood flow, vascularization, and structural changes in large nerve trunks and can objectively confirm nerve thickening in the absence of other cutaneous signs of the disease. Ultrasound can be a non-invasive tool for recognizing neuritis and indicating the need for corticosteroid therapy to prevent permanent nerve damage associated with reactions^5^.

Any nerve trunk or cutaneous nerve can be affected by *M. leprae*, leading to neuropathic manifestations characteristic of the pure neuritic form. The nerves most frequently involved include the supraorbital, great auricular, dorsal cutaneous branches of the radial nerve, and ulnar and superficial peroneal (musculocutaneous) nerve^5^. A study of 70 patients with PNL found that, on average, patients had more than two nerves affected, with the ulnar nerve being the most involved in the upper limbs and the lateral popliteal nerve in the lower limbs, followed by the posterior tibial and sural nerves^1^. Histological series described in the literature have documented alterations in the musculocutaneous nerve between biopsied trunks, consistent with neuritic leprosy, confirming that, although it is less frequently reported than the ulnar or fibular nerve, the musculocutaneous nerve can also be affected in peripheral neuritic leprosy^1^.

Recent studies conducted in Brazil have highlighted the relevance of peripheral nerve ultrasound as a sensitive and reproducible method for early diagnosis of leprosy neuropathy. This allows for the objective detection of nerve thickening and structural changes even before the onset of clinical deficits, enabling the identification of silent neuropathy in household contacts^1^ ^,^ ^10^. Furthermore, the correlation among ENMG, high-resolution ultrasound (US), and laboratory tests reinforced the diagnostic accuracy of pure neural leprosy^1^. 

The study by Voltan et al. (2022), which included the largest Brazilian casuistic study of peripheral nerve assessment in leprosy, reinforces the value of point-of-care ultrasound (POC-USG) as a sensitive tool for detecting neural thickening, particularly of the ulnar nerve in the cubital pretunnel region. The results demonstrated that the ulnar nerve was the only nerve trunk whose variations in ΔCSA and ΔTPT were not influenced by age, which reinforces its importance in the evaluation of leprosy neuropathy. In this context, ΔCSA corresponds to the variation in the nerve cross-sectional area, a parameter used to identify structural neural alterations, whereas ΔTPT refers to the variation in total peripheral nerve thickness, employed to estimate the degree of neural thickening over time or across different segments. However, in the present case, USG could not be performed because of the unavailability of equipment at the referral service. The diagnosis relied instead on dermatoneurological examination, ENMG, and MRI, which together indicated neural involvement compatible with pure neuritic leprosy^10^.

The use of PCR to detect *M. leprae* DNA has been reported as a useful diagnostic tool for all clinical forms of leprosy, including primary neural forms^1^. However, its application remains limited in PNL because nerve biopsy for PCR analysis is an invasive procedure that often yields negative results owing to the low bacillary load of the disease. In this case, PCR was not performed. Nevertheless, the diagnosis was supported by clinical, neurological, and serological findings, which together provided strong evidence of pure neural leprosy. 

Studies have shown that ENMG in patients with leprosy is extremely important as it allows for severity stratification, definition of peripheral neural impairment patterns, and early detection of PNL. Some patients with PNL may present with the subclinical form, in which sensory conduction studies are superior to thermal sensation, vibratory, strength, and monofilament tests in detecting neural impairment at earlier stages^5^. A previous study demonstrated that anti-PGL-I ELISA positivity at diagnosis, in most cases, highlights its importance as a screening and risk indicator, especially in oligosymptomatic patients. However, research has demonstrated that seropositivity does not necessarily indicate active disease in 47.1% of cases and has limited diagnostic value in paucibacillary patients^1^. In this case, other peripheral neuropathies, such as camptodactyly and hereditary neuropathy with pressure susceptibility, were ruled out because of the positive anti-PGL-I test, neurological signs and symptoms of the disease, the patient’s epidemiological history of household contact, and clinical improvement with the initiation of MDT. However, it is important to emphasize that ENMG and ultrasonography are complementary and not diagnostic methods for PNL. Their findings indicate alterations compatible with events resulting from PNL, contributing to the direction of the diagnosis.

The treatment recommended by the Ministry of Health in Brazil is standardized MDT (MDT-U), which comprises a combination of three antimicrobials: rifampicin, dapsone, and clofazimine^4^. This multidrug therapy aims to treat infections; however, it is insufficient to address nerve damage. Prednisone remains the drug of choice for treating neuritis because of its ability to reduce nerve edema, act as an immunosuppressant, and decrease post-inflammatory scarring, all of which are crucial for improving nerve function^11^. 

The use of universal MDT for multibacillary (MB MDT-U) cases in this patient presents challenges as symptoms persisted, and Simplified Neurological Assessment revealed a significant worsening of the neurological condition. Therefore, physiotherapy may be necessary, especially in cases of permanent residual nerve function impairment, as observed in this patient.

In recent years, studies on PNL have advanced significantly. A 2023 study investigated the possibility of a distinct cytokine profile in patients with PNL that differed from other clinical forms of leprosy. Elevated levels of CCL-2 (C-C motif ligand 2 chemokine) have been associated with silent neuritis and correlate with increased levels of IL-10, an anti-inflammatory cytokine^12^. Given the unique nature of PNL, understanding its immunological profile is fundamental to expanding knowledge about the disease and improving diagnostic and treatment strategies. This understanding may provide new perspectives for the clinical management of leprosy and contribute to improving the quality of life of patients with this complex form of the illness. Leprosy is considered the most common peripheral neuropathy of infectious etiology worldwide^1^. The case presented here provides a warning about the importance of neurological signs and symptoms in relation to dermatological findings. 

This case emphasizes the importance of recognizing the diversity of PNL manifestations to improve the diagnosis. Early diagnosis is essential to prevent future injuries, enable appropriate treatment, reduce the risk of severe complications, and improve the patients’ quality of life.
